# Fenced and Fragmented: Conservation Value of Managed Metapopulations

**DOI:** 10.1371/journal.pone.0144605

**Published:** 2015-12-23

**Authors:** Susan M. Miller, Cindy K. Harper, Paulette Bloomer, Jennifer Hofmeyr, Paul J. Funston

**Affiliations:** 1 Department of Nature Conservation, Tshwane University of Technology, Pretoria, Gauteng, South Africa; 2 Veterinary Genetics Laboratory, Faculty of Veterinary Science, University of Pretoria, Onderstepoort, Gauteng, South Africa; 3 Molecular Ecology and Evolution Programme, Department of Genetics, University of Pretoria, Hatfield, Pretoria, Gauteng, South Africa; 4 Veterinary Wildlife Services, South African National Parks, Skukuza, Mpumalanga, South Africa; 5 Lion Program, Panthera, New York, New York, United States of America; University of Illinois at Urbana-Champaign, UNITED STATES

## Abstract

Population fragmentation is threatening biodiversity worldwide. Species that once roamed vast areas are increasingly being conserved in small, isolated areas. Modern management approaches must adapt to ensure the continued survival and conservation value of these populations. In South Africa, a managed metapopulation approach has been adopted for several large carnivore species, all protected in isolated, relatively small, reserves that are fenced. As far as possible these approaches are based on natural metapopulation structures. In this network, over the past 25 years, African lions (*Panthera leo*) were reintroduced into 44 fenced reserves with little attention given to maintaining genetic diversity. To examine the situation, we investigated the current genetic provenance and diversity of these lions. We found that overall genetic diversity was similar to that in a large national park, and included a mixture of four different southern African evolutionarily significant units (ESUs). This mixing of ESUs, while not ideal, provides a unique opportunity to study the impact of mixing ESUs over the long term. We propose a strategic managed metapopulation plan to ensure the maintenance of genetic diversity and improve the long-term conservation value of these lions. This managed metapopulation approach could be applied to other species under similar ecological constraints around the globe.

## Introduction

Biodiversity is threatened worldwide. One of the major threats to biodiversity is habitat loss and resulting population fragmentation. However, population fragmentation is not always as problematic for a population as it may seem, since some species naturally occur as metapopulations (e.g. Melitaeini butterflies [1]). The term metapopulation was first used in by Levins in 1970 in referring to a “population of populations” [2]. Hanski & Gilipin [3] refined the concept in 1991, defining a metapopulation as a “set of local populations which interact via individuals moving among populations”. Harrison [4] realised that classic metapopulations are rare and defined three other types of metapopulation: 1) mainland-island and source-sink metapopulations, 2) patchy populations and 3) non-equilibrium metapopulations. Recently Aycrigg & Garton [5] described the genetic signatures of these four types of metapopulations.

Metapopulation dynamics have been identified in species where human encroachment has caused landscape fragmentation, such as Rocky Mountain elk (*Cervus elaphus nelsoni*) [5], cougar (*Puma concolor*) [6] and lion (*Panthera leo*) [7]. Increasingly it is common for some populations to be completely cut off due to human encroachment, with no natural movement between populations. In extreme cases whole populations have been extirpated from large areas (e.g. African lion in most of South Africa [8]). However, techniques and initiatives have been developed to reintroduce species into patchy, fragmented sections of these landscapes. For example in southern Africa there are numerous examples of large carnivore species being reintroduced into fenced, island-like areas or reserves [9–11]. Ideally in time these fences would be removed allowing conservation areas to expand or wildlife corridors built to link isolated populations. However, this may not always be possible and conservationists must look for innovative ways to manage fragmented populations to maximize their conservation value and ensure their long term existence. Artificial or “managed” metapopulations may be the answer.

“Managed metapopulation” is a recent term in the scientific literature, first appearing with respect to African wild dog (*Lycaon pictus*) management in southern Africa [12]. It has now been used in multiple papers relating to this conservation effort [9,13–15], for cheetah (*Acinonyx jubatus*) management plans [11] and most recently for lions [16]. Managed metapopulations use human-mediated translocations to replace natural movement in areas where habitat is fragmented and no movement is possible due to fencing and/or lack of corridors between populations.

Akçakaya, Mills & Doncaster [17] defined a metapopulation to include the managed metapopulation approach: “a set of discrete populations of the same species, in the same general geographic area, that may exchange individuals through migration, dispersal, or *human-mediated* movement” (emphasis added). They list two requirements for a metapopulation: 1) that the populations are geographically discrete and 2) that mixing between populations is less than within populations [17].

In this paper we explore the potential of a managed metapopulation approach to fenced, fragmented African lion populations in South Africa. This is important because some researchers have questioned the conservation value of reintroducing lions [10]; for although there have definitely been short-term successes [18], their long-term conservation value is uncertain, with some key social systems having broken down due to fragmentation [16].

We propose that a key requirement for conservation success would be for lions from these reserves to be managed in a way such that a) they are genetically similar to the populations that had been extirpated from the region as recommended by the IUCN/SSC Re-introduction Specialist Group [19] b) there are enough prides and adult individuals to form a viable population, and they are managed in such a way that c) simulates natural social mechanisms and d) mimics the genetic diversity and gene flow of a natural metapopulation situation. Björklund [20] indicated that a viable population of lions requires a minimum of 50 prides. This will be met if all lion reserves are managed as a collective [16], thus satisfying the second criterion. Ferreira & Hofmeyr [21] outlined plans to simulate natural social mechanisms that have broken down on small reserves that would satisfy the third criterion. To investigate the genetic provenance, amount of genetic diversity and gene flow that currently exists in the lions, we analyzed DNA samples from fifteen small reserves within South Africa and propose how the lion populations can be managed to satisfy the first and fourth criteria

## Materials and Methods

### Ethics

This project was approved by Tshwane University of Technology Animal Research Ethics board, Pretoria, South Africa (AREC2010/11/004) and the National Zoological Gardens Ethics and Scientific Committee, Pretoria, South Africa (NZG/P12/04). Access to SANParks genetic samples was through a registered project (MILSM964).

### History of Lions in South Africa

Historically lions roamed across most of Africa outside of the Sahara Desert, but their range has contracted markedly and is highly fragmented, and their overall numbers are declining [22,23]. The IUCN Cat Specialist Group developed a regional conservation strategy for eastern and southern African lions in 2006 [24]. Three populations of lions within South Africa were included in this strategy: Kruger National Park (NP), as part of the Greater Limpopo Transfrontier Conservation Area (TFCA); Kgalagadi Transfrontier Park (TP), straddling South Africa and Botswana; and Hluhluwe-iMfolozi Park. More recently, Riggio et al. [22] defined stronghold and potential strongholds of lions across Africa using the population data from the IUCN Cat Specialist strategy [24]. Riggio et al. [22] used the criteria outlined by Björklund [20] for a viable population of lions—a continuous population of a minimum of 50 prides with no limits to dispersal. Only ten large populations across Africa were considered as strongholds including the Great Limpopo TFCA and Kgalagadi TP (Hluhluwe-iMfolzi Park was not evaluated by Riggio et al. [22].) While lions historically roamed across the rest of South Africa, most were extirpated by the early 1900s [8]. Reintroductions of lions into small (<1000 km^2^) public or privately owned fenced reserves have been ongoing since the early 1990s [10,25]. In 2011 there were an estimated 700 lions in 44 reserves (including Hluhluwe-iMfolzi Park) [26]. While the latest 2015 IUCN red list assessment has considered some of these populations [27], they were not considered when continent-wide assessments were conducted in the past [22,24]. This was likely because each population on its own would not have been considered viable. Bauer et al. [23] have recently suggested that lions may increasingly depend on populations in small, fenced, intensively managed reserves in Southern Africa for their continued survival.

Several papers have summarized lion reintroductions into South African reserves [10,16,25]. However, none of them consider genetic origin and date of reintroduction in detail. We have summarised the data from the literature as well as any new data that has been gathered in recent years.

### Microsatellite genotyping

Samples from 351 free-roaming lions in southern Africa were genotyped at 22 microsatellite loci (F42, FCA001, FCA008, FCA026, FCA031, FCA057, FCA075, FCA085, FCA096, FCA097, FCA113, FCA126, FCA193, FCA224, FCA230, FCA240, FCA272, FCA275, FCA391, FCA453, FCA506 and FCA628) as outlined in Miller et al. [28]. Some of these data were previously used in a study to validate these microsatellite loci for use in South African lion population studies [28]. As part of this previous publication, the data were deposited with Dryad (doi:10.5061/dryad.f61vq). We used 301 of the previously genotyped samples in this study along with an additional 50 samples from Tembe Elephant Park, Hluhluwe-iMfolozi Park and Madikwe GR. These new samples were processed as detailed in Miller et al. [28]. The new data set has also been deposited with Dryad (doi:10.5061/dryad.fc2vv). The sampling included material from 17 small reserves, as well as from Kruger NP and Kgalagadi TP, which were included as reference gene pools unaffected by translocations.

As we did not have access to samples from Etosha NP directly, samples from Pilanesberg NP were used as Etosha NP surrogates as this population was established with lions translocated from Etosha NP in the early 1990s [10]. In order to test the validity of this, seventeen samples from lions at Tembe Elephant Park (established in 2001 from Pilanesberg NP and Madikwe NP lions all of Etosha NP decent [10]) were sent to the Leibniz-Institute for Zoo and Wildlife Research in Berlin, Germany. There they were added to a study conducted in collaboration with the African Lion Interest Group of the Felid Taxon Advisiory Group (European Association of Zoos and Aquaria) that included samples from lions in Etosha NP collected by Africat. The preliminary results showed that the samples from Tembe Elephant Park and Etosha NP clustered together (pers. comm. Frank Oberwemmer, August 2015). This, combined with detailed records indicating that no new animals have been introduced into either Pilanesberg NP or Tembe Elephant Park, suggested that the samples from Pilanesberg NP were a realistic surrogate for Etosha NP samples.

**Fig 1:**
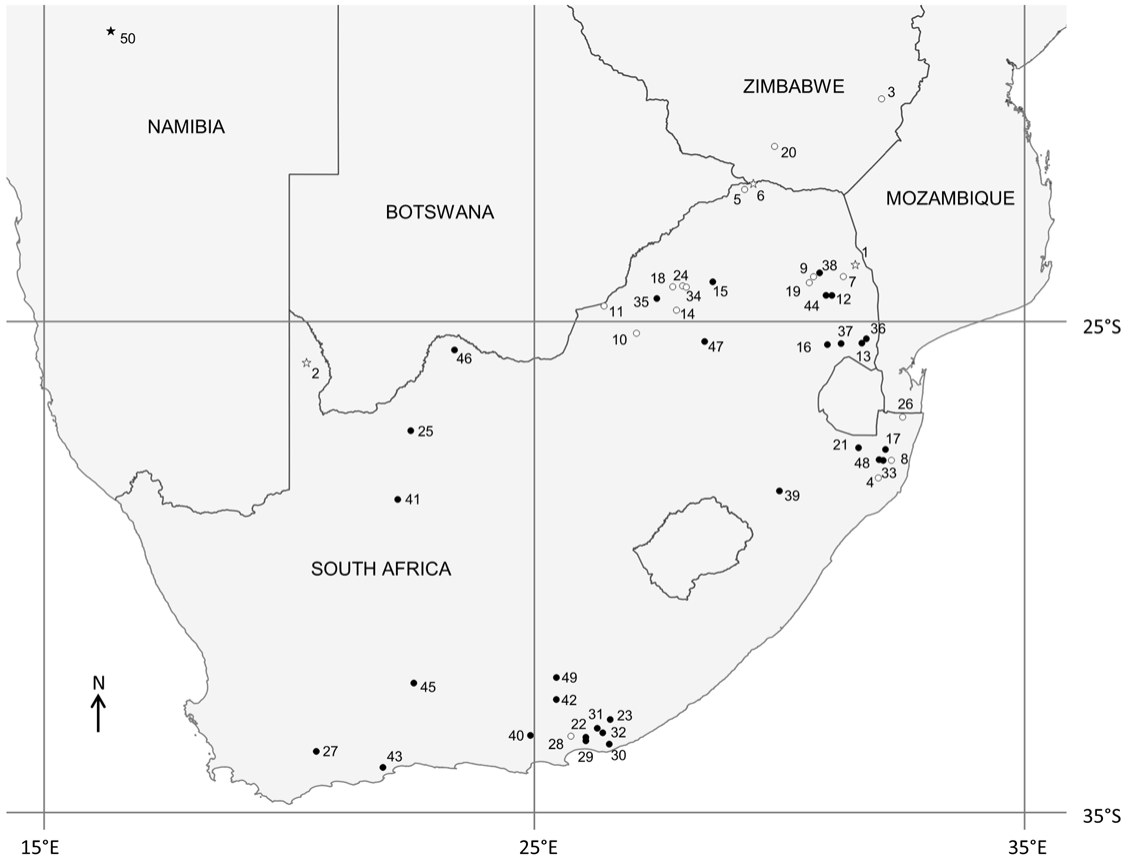
Location of lion populations and sampling sites as outlined in Table 1. Circles indicate reintroduced populations. Stars indicate source populations. Open symbols indicate sampling sites. Modified from Miller et al. 2014 and reprinted under a CC BY license, with permission from Oxford Journals, original copyright (2014).

Samples previously analysed in Miller et al. [28] from Kruger NP and Kgalagadi TP were used to represent those populations respectively. A subset of samples from Venetia-Limpopo NR, along with two samples from Mapungubwe NP, was used to represent the Greater Mapungubwe TFCA lions [28]. Table 1 summarises the origin and number of samples from each reserve and Fig 1 shows the geographic location of each source.

**Table 1:** Reintroduction history including genetic provenance and sampling.

	Reserve	Date of Reintroductions	Genetic provenance of reintroduced lions	Genetic samples for this project
1	Kruger NP		Native	54
2	Kgalagadi TP		Native	11
3	Save Valley Conservancy		Native/Reintroduced	11
4	Hluhluwe-iMfolozi Park	1960s/1999-2001	Kruger/Etosha	63
5	Venetia-Limpopo NR	Formed in early 1990s	Greater Mapungubwe TFCA, Botswana*	16
6	Mapungubwe NP	Formed in 1995	Greater Mapungubwe TFCA, Botswana*	2
7	Thornybush GR	1991	Kruger^†^	4
8	Mun-ya-wana GR	1992/2003/2009	Kruger^†^/Etosha/Kgalgadi	41
9	Greater Makalali PGR	1994/2007	Kruger^†^/Etosha	10
10	Pilanesberg NP	1994	Etosha*	26
11	Madikwe GR	1995	Etosha*	6
12	Kapama PGR	1997	Kruger^†^	-
13	Ligwalagwala GR	1997	Etosha	-
14	Madjuma GR	1997	Etosha	3
15	Entabeni PGR	1998	Etosha	-
16	Lowhills GR	1998	Kruger^†^	-
17	Mkuze Falls GR	1998	Kruger	-
18	Welgevonden PGR	1998/2007	Etosha/Kgalagadi*	31
19	Karongwe PGR	1999	Kruger	4
20	Bubye Valley Conservancy	1999	Native/Etosha	23
21	KwaZulu PGR	2000	Kruger/Etosha	-
22	Shamwari GR	2000	Etosha	-
23	Kwandwe PGR	2001/2008	Etosha/Kruger	-
24	Shambala PGR	2001	Etosha	3
25	Tswalu Kalahari Reserve^a^	2001	Kgalagadi*	-
26	Tembe Elephant Park	2002	Etosha	24
27	Sanbona Wildlife Reserve	2003	Etosha/Kruger	-
28	Addo Elelphant NP	2004	Kgalagadi*	9
29	Amakhala GR	2004	Etosha/Kgalagadi	-
30	Kariega GR	2004	Etosha/Kruger	-
31	Lalibela GR	2004	Mixed	-
32	Pumba GR	2004	Mixed	-
33	Thanda GR	2004	Kruger/Etosha	-
34	Ka'Ingo PGR	2005	Etosha/Kruger	3
35	Marakele NP	2005	Etosha/Kgalagadi*	-
36	Marloth Park	2005	Kruger^†^	-
37	Mthethomusha GR	2005	Kruger^†^	-
38	Selati GR	2005	Etosha/Kruger	-
39	Nambiti Conservancy	2006	Kruger/Etosha	-
40	Blaauwbosch GR	2007	Kruger/Etosha	-
41	Kalahari Oryx	2007/2012	Kgalagadi/Etosha	-
42	Kamala GR	2007	Etosha	-
43	Gondwana GR	2009	Etosha/Kgalgadi	-
44	Blue Canyon Conservancy	2010	Etosha	-
45	Karoo NP	2010	Kgalagadi	-
46	Khamab Kalahari GR	2011	Kgalagadi/Etosha	-
47	Dinokeng PGR	2011	Etosha/Kgalagadi	-
48	Zululand Rhino PGR	2011	Kruger/Etosha Mix	-
49	Mountain Zebra NP	2013	Kgalagadi/Etosha Mix	-
50	Etosha NP		Native	-

^a^Tswalu previously had Kruger origin lions introduced in 1996. They completely replaced them with Kgalagadi origin lions in 2001.

*From original source populations.

^†^From Kruger NP directly, or from neighbouring reserves that are now open with Kruger NP (but were not at the time of translocation)–exact details are not clear for most of these reserves. All other introductions were via another reserve.

### Microsatellite cluster analyses

A subset of the data from unmixed populations (54 Kruger NP, 11 Kgalagadi TP, 26 Pilanesberg NP and 10 Greater Mapungubwe TFCA; total n = 101) was analysed with STRUCTURE [29] to determine the number of genetically distinct groups present in South Africa. K-values from one to seven were tested with a burn-in of 100,000 and data collection of 100,000 chains, with 100 iterations per K-value. The “No Admixture” model was used as no recent mixing was thought to have occurred between any of these populations. STRUCTURE HARVESTER web version 0.6.93 [30] was used to perform the Evanno method [31] for determining the most likely value of K and to prepare the data for input into CLUMPP [32]. CLUMPP was run for all K-values with the “Greedy” algorithm and 1000 repeats to average the results from the STRUCTURE analyses. CLUMPP output files were converted to PS files with Distruct [33]. While the ΔK statistic suggested that K = 2 was the likely number of clusters, the mean of the estimated Ln probability of the data did not level off and there was a small secondary peak at K of four, suggesting some substructure. Therefore, the analysis was repeated for Kruger NP, Kgalagadi TP and Greater Mapungubwe TFCA samples at K = 2 with 100 replicates per value of K.

### Assignment Testing and Levels of Mixing

The data set from reserve animals only (i.e. not including the reference populations) (n = 286) was run in STRUCTURE with K-values from one to eight with 100,000 of burn-in and data collection of 100,000 chains. The “Admixture Model” was applied as some reserves were founded from multiple sources and genealogical records showed subsequent mixing. This was replicated 100 times per value of K. STRUCTURE HARVESTER, CLUMPP and Distruct were used to process the data as above. However the “LargeKGreedy” algorithm was used with 100 iterations within CLUMPP. Samples with a Q value of less than 0.8 were designated as admixed.

### Genetic differentiation and diversity

Number of alleles, percentage of alleles present, allelic richness, observed and expected heterozygosities were determined for each reserve using the ‘divBasic’ command in the R package ‘diveRsity’ [34]. Inbreeding levels (*F*_IS_) were determined using GenePop [35]. Samples from all small reserves (not including HiP) were then combined and compared to Kruger NP, Kgalagadi TP and HiP clusters.

### Relatedness testing for translocation decisions

The program Coancestry was used to determine pairwise relatedness between all individuals [36]. Both the newer Wang [36] and the more traditional Queller and Goodnight [37] algorithms were used. A case study is presented to illustrate the usefulness of relatedness testing when planning translocations. Two males had been moved from Reserve A to Reserve B. The managers then wanted to move two females, also from Reserve A, to join the males on Reserve B. The relatedness of the females to the males was determined in order to inform the managers on the suitability of these individuals for translocation.

## Results

### Reintroduction history

Table 1 and Fig 1 summarise the reintroduction history of lions in small reserves in South Africa. Fig 1 shows the locations of the reserves and the numbers correspond to the numbers in Table 1. Table 1 gives the date of the first reintroduction of lions on each reserve as well as the genetic provenance of those animals. If there was a second genetic provenance introduced, the date of this reintroduction is also listed.

### Cluster analyses

**Fig 2:**
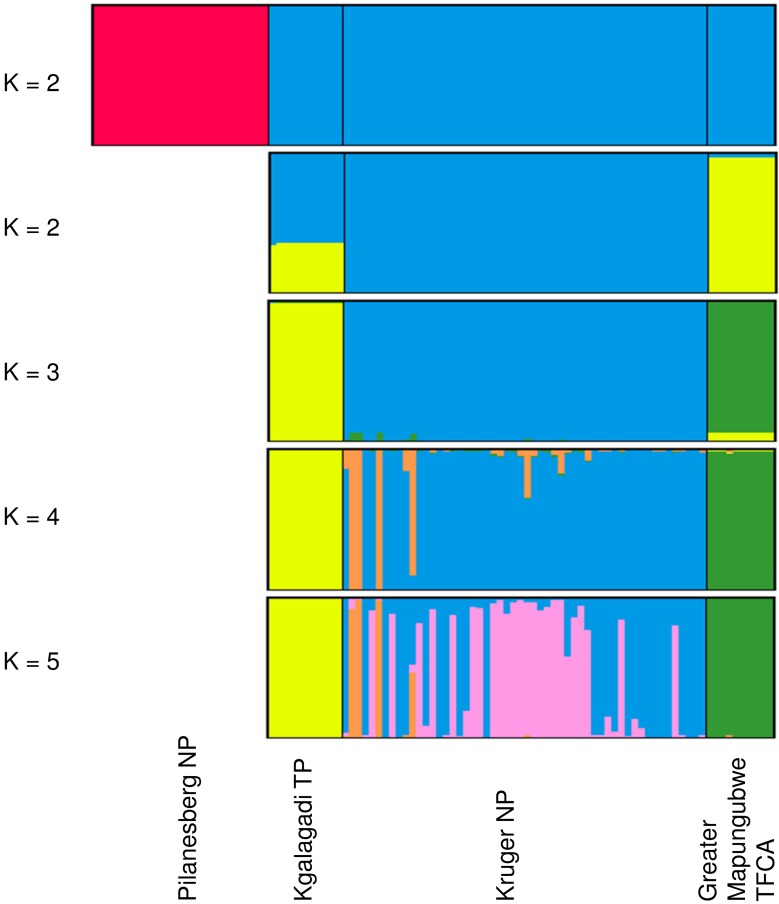
Hierarchical population structure based on STRUCTURE analyses of lion genotypes at 22 microsatellite loci. The first bar chart includes data for samples from Pilanesberg NP, Kgalagadi TP, Kruger NP and Greater Mapungubwe TFCA divided into two clusters (K = 2). The remaining bar charts are the results of the subdivision of the larger of the two clusters from the first analysis for K = 2 to K = 5. Each colour represents the individuals within each cluster. The black lines divide the individuals based on geographical origin of the samples.

**Fig 3:**
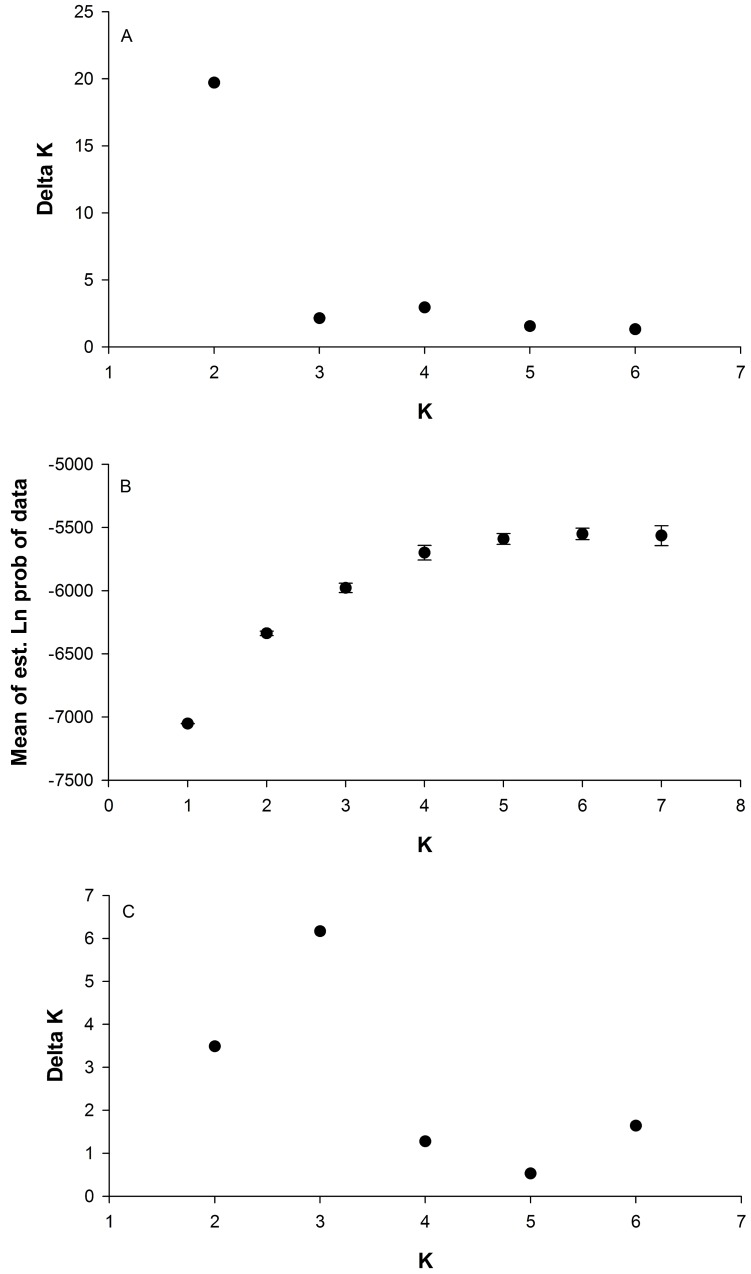
STRUCTURE Harvester output for cluster analysis in Fig 1. a) Evanno ΔK statistic as calculated for K-values of one to seven for STRUCTURE analysis with samples from Pilanesberg NP, Kgalagadi TP, Kruger NP and Greater Mapungubwe TFCA. b) Ln(K) for each value of K with standard deviation error bars. c) Evanno ΔK statistic as calculated for results in Fig 2 for the subclustering (without Pilanesberg NP).

STRUCTURE analyses revealed four clusters that matched the four predicted populations of Kruger NP, Pilanesberg NP, Kgalagadi TP and Greater Mapungubwe TFCA (Fig 2). The Evanno ΔK statistic indicated the strongest population division at K = 2 (Fig 3A) between Pilanesberg NP and the rest (Fig 2). However, the mean of the estimated Ln probability of the data did not plateau until K = 4 (Fig 3B), where there was a small secondary peak in the ΔK statistic (Fig 3A), suggesting there was some finer scale structure [31]. To test this, the analysis was performed without the Pilanesberg NP samples and three further clusters were identified by the ΔK Evanno statistic (Fig 3C). Some splitting within the Kruger NP cluster may exist at higher levels of K (Fig 2).

### Assignment testing

**Fig 4:**
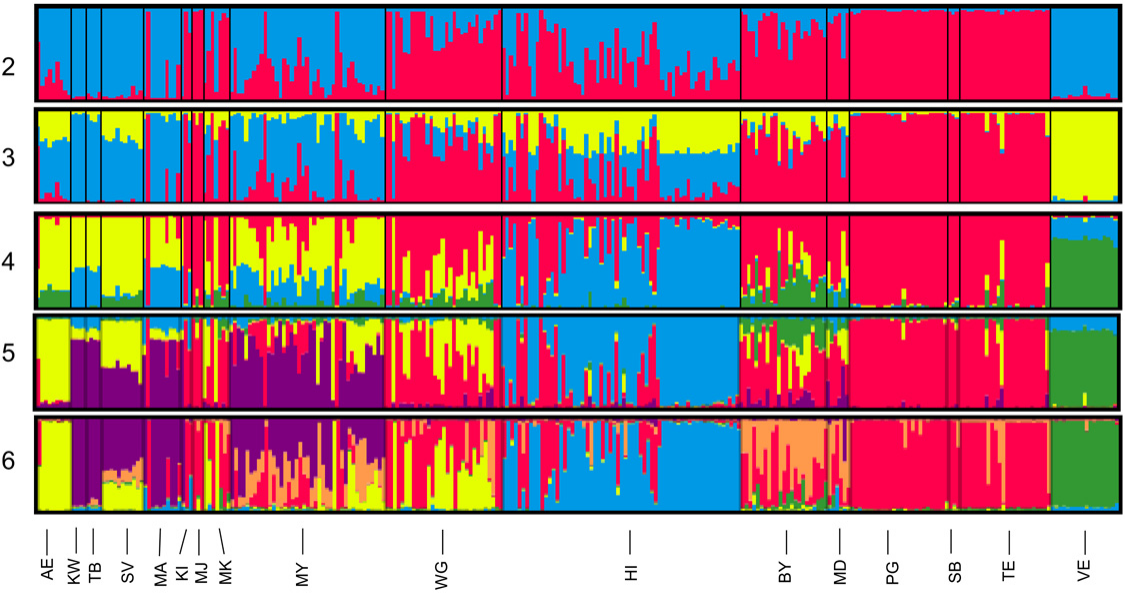
Hierarchical population structure based on STRUCTURE analyses of 22 microsatellite loci. Assignment of individuals from each reserve to clusters; each colour represents a unique cluster. Numbers indicate the value of K. Abbreviations: Addo Elephant NP (AE); Karongwe GR (KW); Thornybush GR (TB); Greater Makalali Conservancy (MA); Ka’Ingo GR (KI); Madjuma GR (MJ); Marakele NP (MK); Mun-ya-wana GR (MY); Welgevonden GR (WG); Hluhluwe-iMfolozi Park (HI); Madikwe GR (MD); Pilanesberg NP (PG); Sanbona GR (SB); Tembe Elephant Park (TE); De Beers Venetia-Limpopo NR (VE): Savé Valley Conservancy (SV); Bubye Valley Conservancy (BY).

The STRUCTURE analysis revealed many reserves to have a mix of sources (Fig 4). Additionally some individual animals were admixed (Fig 4). The analyses also suggested that there were samples within some small reserves that did not fit within the four clusters defined above (Fig 4). Specifically, HiP lions included lions that formed a separate cluster, but recent translocations from Pilanesberg and Madikwe NP (Etosha source) were also evident (Fig 4). Further evidence of some substructure within Kruger NP was indicated by lions from Mun-ya-wana GR that appear to be from a sub-cluster of Kruger NP rather than from the main Kruger NP cluster as seen at a K of six (Fig 4). These lions were originally sourced from Sabi Sand GR which borders Kruger NP. Very little of the clustering associated with Greater Mapungubwe TFCA lions appeared in other lions within South Africa. There was, however, a larger presence of this cluster within some the Bubye Valley Conservancy lions (Fig 4).

### Genetic differentiation and diversity

The allelic richness values were lower in all individual small reserves compared to Kruger NP (Table 2), however, when the small reserves were combined the allelic richness was much closer to the Kruger NP value (Table 3). The small reserve with the highest AR value was Mun-ya-waya GR (Table 2). Mun-ya-waya GR had lions from Kruger NP, Kgalagadi TP and Etosha NP origin (the latter via Pilanesberg NP; Table 1). All of the reserves, except Marakele NP (*F*_IS_ = 0.213), had very low *F*_IS_ values (range -0.18 to 0.073; Table 2). It is possible that the Marakele NP lions that were sampled were closely related (no genealogical data were available). The overall inbreeding level when the small reserves were combined was 0.18 compared to Kruger NP at 0.05 (Table 3).

**Table 2:** Genetic diversity within individual reserves.

	Reserve																		
	KP	KG	AE	KW	TB	MA	KI	MJ	MK	MY	WG	HI	MD	PG	SB	TE	VE	SV	BY
Number of samples	54	11	9	4	4	10	3	3	7	41	31	63	6	26	3	24	18	11	23
Number of alleles	147	81	81	53	56	83	71	51	79	110	105	94	69	76	52	72	64	70	96
Allelic richness	3.12	2.61	2.58	2.16	2.23	2.54	2.65	2.04	2.57	2.94	2.7	2.82	2.37	2.37	2.08	2.16	2.05	2.23	2.62
Observed heterozygosity	0.64	0.55	0.55	0.61	0.62	0.55	0.65	0.55	0.49	0.67	0.57	0.65	0.53	0.53	0.52	0.44	0.39	0.42	0.54
Expected heterozygosity	0.7	0.58	0.59	0.47	0.48	0.57	0.59	0.43	0.59	0.65	0.61	0.63	0.52	0.54	0.44	0.47	0.42	0.52	0.6
Inbreeding coefficient (*F*_IS_)	0.05	0.04	0.03	-0.18	-0.15	0.07	0.09	-0.24	0.21	-0.08	-0.04	-0.03	-0.05	-0.04	-0.09	-0.07	0.03	0.10	0.00

Inbreeding coefficients were calculated using GenePop and the rest of the values were calculated using the R package diveRsity. Abbreviations: Kruger NP (KP); Kgalagadi TP (KG); Addo Elephant NP (AE); Karongwe GR (KG); Thornybush GR (TB); Greater Makalali Conservancy (MA); Ka’Ingo GR (KI); Madjuma GR (MJ); Marakele NP (MK); Mun-ya-wana GR (MY); Welgevonden GR (WG); Hluhluwe-iMfolozi Park (HI); Madikwe GR (MD); Pilanesberg NP (PG); Sanbona GR (SB); Tembe Elephant Park (TE); De Beers Venetia-Limpopo NR (VE): Savé Valley Conservancy (SV); Bubye Valley Conservancy (BY).

**Table 3:** Genetic diversity of lions from South African small reserves combined compared to other populations.

	Kruger NP	Kgalagadi TP	Small Reserves	Hluhluwe-iMfolozi Park	Save Valley Conservancy	Bubye Valley Conservancy
Number of samples	54	11	189	63	11	23
Number of alleles	147	81	155	94	70	96
Allelic richness	4.88	3.42	4.79	3.76	2.93	3.73
Observed heterozygosity	0.64	0.55	0.55	0.65	0.42	0.54
Expected heterozygosity	0.70	0.58	0.71	0.63	0.52	0.60
Inbreeding coefficient (*F*_IS_)	0.05	0.04	0.18	-0.03	0.10	0.00

Inbreeding coefficients were calculated using GenePop and the rest of the values were calculated using the R package diveRsity.

### Relatedness testing for translocation decisions

The case study revealed that one of the males was related at the full sibling level to one female and a half sibling level to the other female, based on both relatedness statistics (Table 4). The decision was made not to move the females.

**Table 4:** Relatedness testing for translocation decisions.

	F2	M1	M2
F1	0.62/0.46	0.50/0.40	-0.06/-0.17
F2	-	0.35/0.22	0.09/0.05
M1	-	-	0.22/0.18

Relatedness results from Coancestry between two female lions (F1 and F2) and two male lions (M1 and M2). The first number is the Wang statistic, the second is the Queller and Goodnight statistic.

## Discussion

The combined genetic diversity of the lions on the fenced, fragmented lion populations in South Africa was similar to that found in Kruger NP suggesting that there is enough genetic diversity within this population to sustain an independent “population” as a managed metapopulation. Their genetic provenances are all southern African, originating in Kruger NP, Kgalagadi TP, Etosha NP and Greater Mapungubwe TFCA, and while Etosha NP provenance is not ideal (see below), the source of lions in reintroductions has been relatively historically accurate. Gene flow has been occurring (through translocations) as evidenced by mixed genetic provenances of many animals and other statistical analyses. All of these combined suggest that the genetic potential of the small reserve lions is enough to satisfy the requirement to be a metapopulation.

Although there was a separate cluster in the HiP lions, this was presumably due to a founder effect as this lion population was started from very small numbers in the late 1950s, early 1960s with no additional founders until the late 1990s and early 2000s when some animals from Pilanesberg NP and Madikwe GR were added to address inbreeding concerns [38]. As this appears to be a recent founder effect, there is no reason to attempt to preserve this genetic cluster.

Kruger NP, Kgalagadi TP, Greater Mabungubwe TFCA and Etosha NP are recognised as separate Lion Conservation Units (LCU) by the IUCN [24] and separate Evolutionarily Significant Units (ESUs) by Barnett et al. [39]. The first three are both located in South Africa and so there is no concern about their presence in the small reserve populations (although mixing may be a concern–see below). Etosha NP provenance may be of concern as Etosha NP is geographically separated from South Africa. While Etosha NP provenance has been found in some populations in Botswana, it has not been found in South African lions outside of the reintroduced populations [40]. This makes sense, as in the past one would have expected some degree of gene flow between Kruger NP, Kgalagadi TP and Greater Mapungubwe TFCA, with much less from Etosha NP (due to its far greater physical distance from these populations). Therefore, the proportion of Etosha NP provenance lions in the small reserves in South Africa may be considered artificially high.

A further complication affecting the genetics of these lions is the mixing of animals from separate ESUs. This is not ideal as it may erode genetic diversity and can lead to a loss of local adaptation [41]. Long-term conservation goals should aim to preserve ESUs [42]. This has not been done for most of the small reserves in South Africa. Decisions for reintroductions were made before the genetic structure of the source populations had been examined, and were based on other factors, such as disease status, the availability of lions for translocation, and limiting inbreeding. Therefore there are currently only a few reserves with unmixed provenance (only from Etosha NP and Kgalagadi TP provenance), the rest being a mix of at least two.

Short of removing all of the lions of mixed provenance and/or of Etosha NP provenance (an impractical and ethically questionable move) there is no way to change the current genetic status of lions in South Africa’s small reserves. Thus we recommend that the current animals be administered as one managed metapopulation. This will result in a new genetic group that is a mix of southern African lions and will provide a useful system for future research into the effects of mixing ESUs. We also recommend that no new animals be sourced from Etosha NP. This will reduce the contribution of Etosha NP genetics, the least geographically accurate group in the metapopulation, over the long term.

This population has not to date been formally managed as a metapopulation, however a Biodiversity Management Plan (BMP) for lions in South Africa is in the final stages of government approval [43]. This BMP advocates for metapopulation management of lions on small reserves and the mimicking of natural systems [43]. Many reserves are already implementing management techniques to mimic natural systems (authors pers. obs.) The genetic diversity found within reserves with lions of one genetic source highlighted the reduction expected within isolated populations with limited founders [44]. Both allelic richness and heterozygosity values were higher in reserves with multiple sources, for example Mun-ya-wana GR, than those with one origin, for example Tembe Elephant Park. There has, as yet, been no impact on reproductive potential of females within small reserves of one origin when prepare to those of multiple origins [26], such as the severe inbreeding detectable in HiP [38], Ngorogoro Crater and the Gir Forest [45]. The levels of heterozygosity on the small reserves were still high in comparison to those of the inbred Gir Forest lions [46] and, apart from one reserve, inbreeding coefficients were low. However, the decrease in allelic richness on some reserves (of one genetic source) may be a warning sign, as the number of alleles is an important measure of long-term evolutionary potential [44]. Inbreeding may not yet be evident due to the few generations since introduction. However, if one looks at the allelic richness of the small reserves combined, it is comparable to that observed in the Kruger NP samples, suggesting that collectively managing the reserves as a metapopulation would result in a genetically diverse population and inbreeding would, presumably, be avoided.

When planning a managed metapopulation approach it is useful to have examples from nature on which to base models. The parallels between natural metapopulations and what we are trying to achieve with the managed lion metapopulation should not be ignored. What we are trying to achieve could be seen as a series of linked patchy populations, such populations being “distributed over a patchy… variable habitat, but in which high rates of dispersal effectively unite the patches into a single demographic entity” [4].

With the implementation of the proposed managed metapopulation plan, there will be four nodes, or patches, around the country [16,21]. There is already uninhibited movement within each reserve. Translocations between reserves within each node will be relatively frequent to simulate dispersal, especially of males, to prevent inbreeding. Less frequent translocations between nodes will simulate longer distance dispersal and simulate more long term gene flow. Whenever possible, genetic samples will be used to assist translocation decisions. This will be especially important for the older reserves that have been the source of many of the younger reserves to ensure that they are not translocating closely related animals back to their reserves.

Aycrigg & Garton [5] defined the genetic signatures of four recognised types of metapopulations. It will be useful to use these definitions in future to see how the managed metapopulation is performing. The current status of the small reserves in South Africa do not match any single metapopulation structure model, but rather appear to be a mix of a nonequilibrium and a patchy metapopulation structure model with variation in heterozygosity levels, allelic richness and relatedness values between parks. There are two types of reserves in the system–those that have mixed genetic provenances (and fit more of the patchy metapopulation structure) and those that have maintained their original genetic provenance (and fit more of a nonequilibrium metapopulation structure). This is a result of the historic management practices, and is less than perfect, but nevertheless existing, template on which we intend to build a managed metapopulation of reintroduced lions in South Africa. It would be relatively simple to expand this model to include reintroduced lion populations in adjacent Mozambique, Namibia and Zimbabwe, or even in countries further away. Lions were recently reintroduced from South Africa’s populations into Majete GR, Malawi [47] and Akagera NP, Rwanda [48]. This trend is likely to continue unless better matched source populations can be accessed.

## Conclusions

With strategic metapopulation management, lions on small reserves in South Africa could form another viable population of lions, containing genetic diversity from all of the remaining lion populations in South Africa and contributing to conservation of lions on the African continent. They do contain a mix of ESUs, which may not be ideal, but this gives conservationists an ideal opportunity to study the impact of mixing ESUs in the long term. It is hoped that the lessons we have learnt from studying these lions in South Africa could be applied to other lion populations across Africa and to other species that are subjected to similar constraints on natural ecological processes.
